# An option for measuring maternal mortality in developing countries: a survey using community informants

**DOI:** 10.1186/1471-2393-10-74

**Published:** 2010-11-17

**Authors:** Siti Nurul Qomariyah, David Braunholtz, Endang L Achadi, Karen H Witten, Eko Setyo Pambudi, Trisari Anggondowati, Kamaluddin Latief, Wendy J Graham

**Affiliations:** 1 Centre for Family Welfare, Faculty of Public Health, University of Indonesia, Depok, West Java, 16424, Indonesia; 2 Immpact, School of Medicine and Dentistry, University of Aberdeen, UK

## Abstract

**Background:**

The maternal mortality ratio (MMR) remains high in most developing countries. Local, recent estimates of MMR are needed to motivate policymakers and evaluate interventions. But, estimating MMR, in the absence of vital registration systems, is difficult. This paper describes an efficient approach using village informant networks to capture maternal death cases (Maternal Deaths from Informants/Maternal Death Follow on Review or MADE-IN/MADE-FOR) developed to address this gap, and examines its validity and efficiency.

**Methods:**

MADE-IN used two village informant networks - heads of neighbourhood units (RTs) and health volunteers (Kaders). Informants were invited to attend separate network meetings - through the village head (for the RT) and through health centre for the kaders. Attached to the letter was a form with written instructions requesting informants list deaths of women of reproductive age (WRA) in the village during the previous two years. At a 'listing meeting' the informants' understanding on the form was checked, informants could correct their forms, and then collectively agreed a consolidated list. MADE-FOR consisted of visits relatives of likely pregnancy related deaths (PRDs) identified from MADE-IN, to confirm the PRD status and gather information about the cause of death. Capture-recapture (CRC) analysis enabled estimation of coverage rates of the two networks, and of total PRDs.

**Results:**

The RT network identified a higher proportion of PRDs than the kaders (estimated 0.85 vs. 0.71), but the latter was easier and cheaper to access. Assigned PRD status amongst identified WRA deaths was more accurate for the kader network, and seemingly for more recent deaths, and for deaths from rural areas. Assuming information on live births from an existing source to calculate the MMR, MADE-IN/MADE-FOR cost only $0.1 (US) per women-year risk of exposure, substantially cheaper than alternatives.

**Conclusions:**

This study shows that reliable local, recent estimates of MMR can be obtained relatively cheaply using two independent informant networks to identify cases. Neither network captured all PRDs, but capture-recapture analysis allowed self-calibration. However, it requires careful avoidance of false-positives, and matching of cases identified by both networks, which was achieved by the home visit.

## Background

The maternal mortality ratio (MMR) remains high in most developing countries [1]. Local, recent estimates of MMR are needed to motivate policymakers to prioritise maternal health, and to evaluate interventions. But estimating MMR, in the absence of vital registration systems, is difficult. Large surveys are required for even moderately precise estimates of MMRs [2]. Consequently, such surveys are not undertaken frequently, only provide national level figures, and use long 'reference' periods of 5 or more years; an example for this is the Demographic Health Survey (DHS) used by the Indonesian Government to estimate national MMR [3,4]. What policy makers and program managers need is a relatively cheap and reliable method to provide sub-national-level estimates of recent maternal death rates. This paper describes an approach developed to address this gap.

In Serang and Pandeglang districts, Banten Province, as in all of Indonesia, the administrative system includes community level volunteers (RTs) responsible typically for 10-40 house-holds. Distinct from this are health volunteers (Kaders) responsible for the integrated health posts with coverage of approximately 100 households. Since these systems are comprehensive, functional, and extend down to a level at which it would be reasonable to expect good knowledge of deaths and maybe circumstances of death, there is an obvious possibility of using them as a basis for 'capturing' maternal deaths.

An informant-based method for identifying maternal deaths - Maternal Deaths from Informants/Maternal Death Follow on Review (MADE-IN/MADE-FOR) - was developed and tested by Immpact (Initiative for Maternal Mortality Programme Assessment) in the two districts. It formed a key part of a larger study looking at the effects of the Indonesia village midwife programme [5]. This method was developed as an alternative to allow measurement of maternal mortality down to community level, along with analysis on the cause of death. This paper is written by some of the researchers who involved in the development, as well as pilot and implementation of the method.

MADE-IN uses RT Heads and Kaders as informants who provide information about deaths to women in their communities. Once probable maternal deaths are identified by these informants, the MADE-FOR survey follows up with visits to the families to confirm details and circumstances of the death. This paper describes the method, and examines its validity and efficiency.

## Methods

### Study sites

The study was conducted in all 708 villages in Serang and Pandeglang Districts. In Serang, 38 sub-district health centres (HCs) provide services for almost 1.9 million people, similarly 34 HCs in Pandeglang, with about 1.1 million populations [6]. Banten Province ANC coverage is about 86% but skilled attendance at birth is only about 52% [4].

### Maternal death definition

In this study, as for instance in DHS studies, the MMR is estimated using information on the number of Pregnancy Related Deaths (PRD) [7]. A PRD is defined as the death of a woman while pregnant or within 42 days of termination of pregnancy, irrespective of the cause of death. This category of case is introduced to facilitate identification of maternal death in circumstances in which attribution of cause might be inadequate. From verbal autopsies, we found that the proportion of women classified as PRD who died from maternal causes in the study area was very high (i.e. 97.5%) meaning that choice of PRD or maternal death definition is not of major importance.

### Timeframe

The pilot and data collection process ran from January to June 2006.

### Pilot phases

The pilot phases were aimed at examining the advantages and disadvantages of various possible informant networks, and of different modes of accessing the informants' knowledge. A further feasibility study was conducted to assess the logistics and costs of a full-scale survey, using the most efficient administrative and data capture approaches identified from the pilot.

Kaders were chosen as forming the best network for identifying maternal deaths: they are established in the community and registered both in HCs and sub-district offices, making them easier to contact; their attendance rate was high (on average 90% compared to 52% for RTs), they were able to understand and fill-out the forms with a low error rate (5% compared to 9% for RTs); and are typically one third as many kader as RTs in a village, which reduced costs of data capture. However, because population density is much greater in urban areas and knowledge of neighbours less, it was felt many PRDs might be missed by kaders, and so it was decided to also include the RT network in urban areas. To enable capture-recapture estimates, RTs were also used in one randomly-chosen village per sub-district in rural areas.

### THE 'MADE-IN' PART OF DATA CAPTURE

The key activity was the 'listing-meeting' of village informants, to list details of deaths of women of reproductive age (WRA) in the village. Note that in villages where both kader and RT networks were used, separate listing meetings were held for each network, allowing for independency between the networks. Prior to the meetings, information, instructions, and forms were distributed to the village informants, for them to list information about WRA deaths during the previous two years. The most important items requested on the form were timing of death in relation to any pregnancy, age of woman, date of death, residence of woman, and name and address of a relative. Informants were asked to bring the completed forms to the listing meeting, where the study and forms were explained verbally, questions answered, and an opportunity given for informants to correct their forms in the light of improved understanding. The meeting then discussed all deaths listed by any of the informants, and collectively agreed a 'consolidated' list of WRA deaths, and of likely PRDs. To strive for total coverage of deaths, the MADE-IN process also included a visit to village informants who did not attend the meeting.

### The 'MADE-FOR' part of data capture

**Figure 1:**
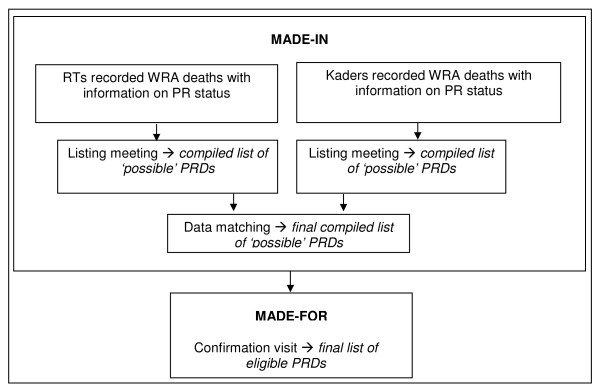
**MADE-IN/MADE-FOR flow of data capture**.

This consisted of visits to the named relative of likely PRDs on a consolidated MADE-IN listing meeting list. This was primarily to confirm or correct the details on the form. For 'eligible' deaths (i.e. PRDs within the defined period, and district), additional information about the circumstances and cause of death were collected through structured verbal autopsy of specific variables to allow estimation of cause of death using a computerised algorithm InterVA-M [8]. Socio-economic status and information on use of health services during pregnancy and near the time of death were also collected. Together these two steps - MADE-IN and MADE-FOR - provided village level estimates of the number of deaths of WRA, and in particular of PRDs in a defined period. Further details about the method are available at http://www.immpact-international.org/toolkit/module4/mimf/index.html. The method is summarised schematically in the following diagram (Figure 1).

### Sources for information on number of live births

Many sources can be used to provide estimates of live births by district. In Indonesia they include Demographic and Health Surveys, National Socioeconomic Surveys or censuses, and District Basic Health Research surveys. However, to enable useful comparisons between PRDs and all women with recent pregnancies, a stratified cluster random sample survey was conducted by Immpact, not only to estimate live births but also to describe characteristics and health status, among other variables. With some help with the sampling, such a survey is well within the capability of a team undertaking MADE-IN MADE-FOR. Villages were stratified as urban, rural or rural-remote. A total of 125 villages were sampled with probability proportional to size and with replacement. Within each sampled village, all household members were listed to identify all women with a live birth or stillbirth between April 2004 and March 2006. Eight births per village were then randomly selected, and information was collected from the families on the same background variables as for maternal deaths.

### Ethical approval

Ethical consideration was one of several issues ensured in this study. It includes permission, confidentiality, and research benefit for the community. Before the study conducted, ethical approval was sought and then issued by the Ethical Committee of Faculty of Public Health University of Indonesia. Permission was also obtained from related district health offices. At community level, permissions were obtained from all sub-districts and villages in the two districts. Before an interview was conducted, written consent was asked from each respondent after data collectors explained the objective, benefit, and process of the interview. All individual data was treated in a strictest confidentiality and no information on name was disclosed, even to the village or sub-district authority. Following the study, researcher provided feed back to various parties from various levels to make sure that public will achieve benefit from the study conducted. Feed back has been continuously given through written dissemination (publication) or seminar, at local, national, and international level.

### Limitation of the study

The main limitation of the method deals with recall of the event. This method relies on informants' memories and knowledge to record deaths, thus the accuracy of the method depends on the length of recall period and size of the area covers by informants. This recall issue also related with the memory of the family in describing the circumstances of the death. Further detail about limitations of the method is available at http://www.immpact-international.org/toolkit/module4/mimf/index.html.

### Analysis

#### Estimating total maternal deaths and risk

In villages where both kader and RT networks were used, for each confirmed eligible PRD we know whether the kader, or RT, or both networks identified the case. This allowed us to use 'capture-recapture' techniques [9] to estimate both the total PRDs in these villages, and the coverage of each network (i.e. the proportion of the total identified by each network). A simple formula which can be used to estimate the total cases is T = (N1*N2)/M. N is number of cases captured by method 1 or method 2 (in this case by RTs or by kaders). M is the total cases captured by both methods. The estimated coverage of the kader network, and actual number of PRDs the kader identified, were then used to estimate the total deaths in the villages where only kaders were used.

It should be noted that although we used inter-VAm to interpret cause of death using information from the MADE-FOR, to calculate the MMR we used PRD definition for the numerator. Reasons for this include the experimental nature of InterVA-M, the very low proportion of non-maternal cases, and also ensure full comparability with the national level estimate derived from the Demographic and Health Survey

The name 'capture re-capture' derives from wildlife applications, where a sample of animals from a target population is captured, marked and released. A second sample is captured at some later time. The number of animals captured each time, and both times, is noted [9]. In public health applications, it is usually individuals who are 'captured' on different databases, and a key stage is matching i.e. identifying the individuals who appear on more than one database.

There are four critical assumptions made in the simple capture-recapture analysis which we have used:

1) The set of 'individuals or 'events' to be estimated is fixed. This is often problematic when estimating total population in animal studies (since they reproduce, migrate, and die - so the individuals, and indeed the total number, changes between capture & recapture), but not in this study since there clearly is a fixed set of PRDs in the defined period and area which are captured, and recaptured by the two networks.

2) Individuals captured by both databases can be matched. This is often a major problem for public health applications, but here this was largely solved via the follow-up visits, where it quickly becomes clear if two apparently different deaths reported by the networks are in fact the same woman.

3) Capture in the second sample is independent of capture in the first. In this study, this is not entirely the case, since despite attempts to keep the network listing processes entirely separate, there were inevitably occasions for 'contamination'. For example, it was not uncommon for RTs to be married to a kader. However, by holding separate meetings, and limiting time available for 'crosschecking, it is believed contamination was very limited. In fact, checks were made on completed forms (looking for similar order, spellings etc) and no evidence of information-sharing came to light.

4) Within each occasion or database, the probability of capture does not differ between individuals. This assumption is problematic, since there are certain types of deaths which tend not to be reported by either network, for example deaths in early pregnancy.

The direction of bias due to assumptions 3 and 4 not being completely met in this study is, in both cases, downwards - so the resulting estimate will tend to be lower than the true number of deaths.

To estimate live-births (to allow an estimate of MMR), fertility-rates from the survey mentioned above, undertaken in the same districts, were applied to the census population estimates. Data processing was undertaken using Epi-info [10], with estimation undertaken in WinBUGS [11] to allow description of uncertainty around estimates.

#### Analysis of cost

The number of PRDs identified per "survey effort" (data-collector-weeks) is one measure used to compare MADE-IN/MADE-FOR with other methods for identifying maternal deaths. Another measure used is "cost per woman-year of exposure." Exposure is calculated from the number of WRA in the area, multiplied by the number of years of exposure. Costs were divided between MADE-IN or MADE-FOR, either as they fall clearly to one (such as informants' transportation fee, which is a MADE-IN expenditure), or, for more general expenditure (wages, accommodation for the survey team), based on the number of data-collector-days spent on MADE-IN and MADE-FOR, which was generally similar. Information on the cost of population survey, of which the estimate of live births is used, is illustrated as unit cost for capturing a live birth.

## Results

### Probability of each informant network capturing deaths

In urban and in 10% of rural villages, where we used both kader and RT networks to capture eligible (i.e. within the set period, and location) PRDs, 116 eligible PRDs were identified: 13 were identified only by the Kader network, 30 identified only by the RT network, and 73 identified by both. This gives a point estimate of 0.71 for the probability of an eligible PRD being identified by MADE-IN/MADE-FOR via Kader information, and an estimate of total eligible PRDs of 121. In other words, there were an estimated 5 extra eligible PRDs in 2004/5 in the areas with both RT and Kader informants, which were not identified by MADE-IN/MADE-FOR (see table 1).

**Table 1:** The probability of the Kader and RT networks in capturing deaths

	Calculation	Result
**In area with kaders and RTs meeting**		
Number of cases captured by RT	-	103
Number of cases captured by Kaders	-	86
Number of match cases (captured by both)	-	73
Total death cases captured by the two networks	-	116
Simple estimate of total number of PRDs	86 × 103/73	121
Simple estimate of probability of kaders capturing cases	73/121	0.71
Simple estimate of probability of RTs capturing cases	103/121	0.85
Number of cases missed by the networks	121-116	5
**In area with kaders only meetings**		
Number of cases captured by Kaders	-	353
Estimate number of PRDs	353/0.71	498
**In the whole area**		
Mean estimated number of PRDs (with 95% CI) - allowing for uncertainty in the simple estimates above.		627 (560 to 711)

Overall, we estimated that the RT network was more likely to capture PRDs than the kader network. This does not, however, mean that individual RTs are necessarily 'better' than individual kaders in identifying PRDs, since within the same area the number of RTs involved is much higher than the number of kaders.

### The accuracy of reported eligible PRD status of identified WRA deaths

From the pilot phases dataset, when all identified WRA deaths were visited, it was found that the village informants mis-classified 2 out of 25 eligible PRDs as not being eligible PRDs. On the other hand, 8 out of 31 deaths village identified by village informants as being eligible PRDs were false positives. Table 2 below shows the accuracy of the village informants in reporting eligible PRD status, by year and by area, using the MADE-FOR visit result as the "gold standard". The table shows that the kader network is generally more accurate in reporting eligible PRD status. There is a trend towards the eligible PRD status of more recent WRA deaths, and WRA deaths from rural areas, being more accurately reported.

**Table 2:** The accuracy of assigned PR status of identified WRA deaths (MADE-IN process), by informant, year of death, and district*

	N	Sensitivity ^@^	Specificity^β^	PPV	NPV
**Total**	189	92 (23/25)	95.1 (156/164)	74.2 (23/31)	98.7 (156/158)
**By informant**					
Kader	141	95.5 (21/22)	95.8 (114/119)	80.8 (21/26)	99.1 (114/115)
RT	156	91.3 (21/23)	94.7 (126/133)	75 (21/28)	98.4 (126/128)
**By year**					
2004 deaths	88	83.3 (10/12)	92.1 (70/76)	62.5 (10/16)	97.2 (70/72)
2005 deaths	101	100 (13/13)	97.7 (86/88)	86.7 (13/15)	100 (86/86)
**By area**					
Urban	85	88.9 (8/9)	92.1 (70/76)	57.1 (8/14)	98.6 (70/76)
Rural	104	93.8 (15/16)	97.7 (86/88)	88.2 (15/17)	98.9 (86/87)

* Selected only for cases during pilot phases when all WRA deaths were visited; year of death were 2004 or 2005, villages with both kaders and RTs meeting and result of visit was 'complete'

@ @ The denominator in 'sensitivity' is the true number of eligible PRDs, amongst all the 'WRA deaths to residents in the specified period' listed by the informants. The numerator is the number of these who were identified in the informant list as likely to be PRD.

β The denominator in 'specificity' is the true number of non-PRDs, amongst all the 'WRA deaths to residents in the specified period' listed by the informants. The numerator is the number of these who were indentified in the informant list as likely to be non-PRD

### Efficiency of the method

#### Total cost of MADE-IN and MADE-FOR

The study took approximately 18 weeks. To collect the data from all villages in the two districts, 436 person-weeks were consumed. In total the study cost $154,271 (see table 3). This includes salary for field workers, travel cost, supplies and services, administration and capital expenditures. Informants were only given a transportation fee to attend the meeting and provided with a light meal. This is considered appropriate for the Indonesian setting. RTs and kaders are volunteers, and are used to being involved in many community activities without any official payment.

**Table 3:** MADE-IN and MADE-FOR costs, Serang and Pandeglang Districts 2004-2006

	MADE-IN	MADE-FOR	Total
Administration	2,766	3,195	5,961
Salary for field workers	26,629	26,629	53,260
Supplies and service	8,656	5,539	14,195
Travel	45,147	32,966	78,118
Capital expenditures	1,372	1,372	2,745

Total $*	84,571	69,700	154,271

Percentage	**54.8**	**45.2**	
# of WRA	758,000	758,000	758,000
years	2	2	2
Women-year risk of exposure	1,516,000	1,516,000	1,516,000

**cost per women-year risk of exposure ($)**	**0.056**	**0.046**	**0.102**

* $1 = Rp 9000

The total cost for the population survey was $ 130,435. This provided an estimate of the number of live births, but also information on locations and other potential 'risk factor' variables for pregnancy and delivery outcome (e.g. age, parity, socio-economic status, prenatal care status, utilization of health provider for pre-natal and intra-partum care). This information was used together with the information on PRDs, to estimate relative risks. We believe that in most settings where MADE-IN/MADE-FOR could be used, information on live births will be available from other existing sources, so that a population survey is an optional, but extremely valuable, extra not strictly required for estimating MMR.

#### Added value from the MADE-FOR step

If only information from the MADE-IN process (without the verification visit of MADE-FOR) is used, some 'matches' (PRDs reported in both networks) are missed, and some 'false positives' remain undetected. The resulting capture-recapture analysis over-estimates the number of PRDs captured, and under-estimates the probability of the kader network capturing PRDs at 0.53, and consequently severely over-estimates the total number of deaths, and the MMR, as seen in Table 4.

**Table 4:** Comparison of MMR estimates in the two districts and total cost of data collection

Method	Total cost*	Cost per death ($)	MMR estimates	CI
**MADE-IN with CRC**	84,571	0.056	731**	690 - 780
**MADE-IN/MADE-FOR with CRC**	154,271	0.102	434**	377 - 499
Indonesia***				
1. IDHS (2002/2003)		12	307	-
2. IDHS (2007/2008)			228	-
3. WHO (2005)			420	240-600

* assuming the additional cost for CRC calculation is very low

** Estimates generated for the two districts

*** Sources: IDHS 2002/2003 [8], IDHS 2007/2008 [3], WHO: 2007 [1]

While MADE-FOR increased costs by about 50%, without this step bias due to unrecognised false positives, and missed matches, is so large as to make the estimates of PRDs and MMR, almost worthless. The size of this bias is unlikely to be stable across time or region, as it depends on the operational characteristics of the informant networks, so any 'adjustment factor' to convert a MADE-IN only estimate to an unbiased one, will be subject to great uncertainty. As well as verifying the status of PRDs, MADE-FOR allows collection of information about the circumstances of death, and characteristics of the deceased. This enables assessment of risk factors, and causes of death, which are invaluable for service planning.

## Discussion

Currently, Indonesia relies primarily on a national MMR estimate generated from the IDHS in 2008. The design and size of the IDHS sample meant only 62 PRDs, over a 5 year 'window', were found, and hence a very imprecise estimates even at national level. Given the diversity of Indonesia in terms of health services and health profile, this national average is likely to conceal more than it reveals, and because it is averaged over the last five years it will not capture recent changes. The lack of precise, local, and current estimates of maternal mortality in Indonesia is a major problem when trying to assess local needs, or the impact of programmes to reduce maternal risks - which was one motivation for developing MADE-IN/MADE-FOR. But it is also a major problem when it comes to assessing the validity of any measurement method, including MADE-IN/MADE-FOR. We have assessed the reliability, feasibility and efficiency of MADE-IN/MADE-FOR using internal consistency checks, and assurance of high quality data capture processes.

The key characteristic of MADE-IN/MADE-FOR is the use of existing networks of village informants to report vital events retrospectively. In combination with existing information on population and fertility, this allows a precise, local estimate of MMR to be obtained quickly from a 'one-off' survey. A companion 'population survey' allows investigation of risk factors, but increases costs. There is clearly the potential to develop the process into a sustainable ongoing system, which might result in higher quality data since events would be more recent - for instance verbal autopsy interviews would always be conducted within a reasonably short period of the deaths. Many countries in the region have also used villagers in health programs including in recording and reporting systems. A study in Cambodia, for example, involved Village Health Volunteers in a community-based surveillance system [12], and found that it could successfully fill the gaps of the current health facility-based disease surveillance system. A similar method has also been used in India [13].

The use of two informant networks in the MADE-IN/MADE-FOR is an important innovation, as it allows capture re-capture estimation. This makes the survey 'self-calibrating' in that it can estimate its own coverage, in a situation where it is clear that no single method captures all deaths. A study in Cambodia which tried to compare two surveys methods (a community based survey and a household survey) for estimating maternal and perinatal mortality found detection failures in both surveys, as high as 30-40% [14]. A study in the USA found only 62% of maternal deaths were identified through death records [15].

The huge advantage of MADE-IN/MADE-FOR over household surveys like DHS, is that costs are reduced by a factor of 10-100. For the DHS, the level of investment required to ensure a high-quality survey, such as long periods of training, extensive pilot testing, separate household listing teams prior to the survey, and maintaining data quality tables during fieldwork, along with considerable technical assistance needed, can result in costs easily exceeding US$150 per household interview [16], implying $12 or more per woman-year of exposure (calculated from mean number of WRA in a household from the Indonesia DHS which is 2.4 and a 5 year 'window'), while MADE-IN/MADE-FOR only cost $0.1 per woman-year of exposure. It should, however, be noted that the DHS collects much more information than MADE-IN/MADE-FOR.

This two step method is very similar to the rather loosely defined RAMOS (reproductive age mortality study) method which is often regarded as the gold standard for estimating maternal mortality in developing countries, if conducted properly [17-19]. Both methods start by identifying deaths to WRA together with information on time of death related to pregnancy status. The difference is that the first step of RAMOS uses all available methods - existing records (e.g. vital registration, health facilities reports, burial records), and any appropriate types of informants, to gather data on deaths to WRA. MADE-IN/MADE-FOR in contrast, restricted itself to using Kader and RT informants only - to keep costs down. The second step of RAMOS, investigating the cause of death to WRA, is very similar to MADE-FOR, except the latter only involves visits to probable PRDs. The MADE-FOR step is crucial in verifying PRDs, as otherwise false-positives and undetected matching cases severely inflate the capture-recapture MMR estimate.

## Conclusions

This study shows that reliable local, recent estimates of maternal mortality can be obtained quickly and relatively cheaply using two independent informant networks to retrospectively identify cases. Capture-recapture estimation allows self-calibration, but requires avoidance of false-positives, and that matches are identified, which in this study was achieved by means of the visit to interview a relative of the dead women. The visit was also used to investigate cause of death and risk factors.

A similar approach might be used prospectively, although this is organisationally a very different task. Such a system might even be used as basis for a reliable vital registration system. Ultimately, Indonesia and all other countries in the world must develop reliable civil registration and health management information systems to provide the necessary data for monitoring and evaluation of health programs including reproductive health.

## Competing interests

The authors declare that they have no competing interests.

## Authors' contributions

SNQ led the data collection process in the field, contributed substantially to the analysis, and undertook primary responsibility for all aspects of the manuscript; DB contributed substantially to the design of the study and revision of the draft, and led the analysis; ELA conceived in the original idea, contributed to the revision of the draft; KHW contributed substantially to the design of the study, data collection, and data processing; ESP contributed substantially to data collection, and led data processing and database maintenance; TA contributed substantially to data collection and helped with data-processing and analysis; KL contributed substantially to data collection, and helped with data-processing; WJG contributed substantially to the revision of the draft. All authors read and approved the final manuscript.

## Pre-publication history

The pre-publication history for this paper can be accessed here:

http://www.biomedcentral.com/1471-2393/10/74/prepub
